# Mitochondria dysfunction in CD8+ T cells as an important contributing factor for cancer development and a potential target for cancer treatment: a review

**DOI:** 10.1186/s13046-022-02439-6

**Published:** 2022-07-21

**Authors:** Lu Zhang, Wen Zhang, Ziye Li, Shumeng Lin, Tiansheng Zheng, Bingjie Hao, Yaqin Hou, Yanfei Zhang, Kai Wang, Chenge Qin, Liduo Yue, Jing Jin, Ming Li, Lihong Fan

**Affiliations:** 1 Department of Respiratory Medicine, Shanghai Tenth People’s Hospital, Tongji University School of Medicine, Shanghai, 200072 China; 2 Shanghai Tenth People’s Hospital, Anhui Medical University, Hefei, China; 3 Clinical Medical College of Shanghai Tenth Hospital of Nanjing Medical University, Nanjing, China; 4 Medical School of Nantong University, Nantong, Jiangsu 22601 People’s Republic of China; 5 Institute of Energy Metabolism and Health, Tongji University School of Medicine, Shanghai, China; 6 Integrated Chinese and Western Medicine Pulmonary Nodules Center, Shanghai Tenth People’s Hospital, Tongji University School of Medicine, Shanghai, China

**Keywords:** Mitochondria, CD8+ T cells, cancer development, Anti-tumor immunity, cancer treatment

## Abstract

CD8+ T cells play a central role in anti-tumor immunity. Naïve CD8+ T cells are active upon tumor antigen stimulation, and then differentiate into functional cells and migrate towards the tumor sites. Activated CD8+ T cells can directly destroy tumor cells by releasing perforin and granzymes and inducing apoptosis mediated by the death ligand/death receptor. They also secrete cytokines to regulate the immune system against tumor cells. Mitochondria are the central hub of metabolism and signaling, required for polarization, and migration of CD8+ T cells. Many studies have demonstrated that mitochondrial dysfunction impairs the anti-tumor activity of CD8+ T cells through various pathways. Mitochondrial energy metabolism maladjustment will cause a cellular energy crisis in CD8+ T cells. Abnormally high levels of mitochondrial reactive oxygen species will damage the integrity and architecture of biofilms of CD8+ T cells. Disordered mitochondrial dynamics will affect the mitochondrial number and localization within cells, further affecting the function of CD8+ T cells. Increased mitochondria-mediated intrinsic apoptosis will decrease the lifespan and quantity of CD8+ T cells. Excessively low mitochondrial membrane potential will cause the release of cytochrome c and apoptosis of CD8+ T cells, while excessively high will exacerbate oxidative stress. Dysregulation of mitochondrial Ca2+ signaling will affect various physiological pathways in CD8+ T cells. To some extent, mitochondrial abnormality in CD8+ T cells contributes to cancer development. So far, targeting mitochondrial energy metabolism, mitochondrial dynamics, mitochondria-mediated cell apoptosis, and other mitochondrial physiological processes to rebuild the anti-tumor function of CD8+ T cells has proved effective in some cancer models. Thus, mitochondria in CD8+ T cells may be a potential and powerful target for cancer treatment in the future.

## Background

Cancer remains the major cause of death even in wealthy countries [1]. T-cell-mediated cellular immune response is known to play a crucial role in anti-tumor immunity. Many previous studies have demonstrated a positive correlation between a high level of T-cell infiltration and a favorable prognosis in breast, lung, ovarian, colorectal, renal, prostate and gastric cancers, and melanoma as well [2]. Mature T cells are categorized into two subgroups: CD4+ and CD8+. Compared with CD4+ T cells, CD8+ T cells are more prominent anti-tumor cells in the body [3, 4].

When stimulated by processed tumor antigen-derived peptide, resting CD8+ T cells are activated via the interaction between T cell antigen receptor (TCR) -CD3 complex and major histocompatibility complex (MHC) I molecule. Simultaneously, costimulatory molecules, CD8 and CD28 provide the second signals for the reaction [5]. Cytokines such as interleukin 2 (IL-2), IL-4, IL-15 and IL-7 are consumed to maintain the later proliferation of activated CD8+ T cells [6, 7]. Upon co-stimulation from CD80, CD70, 4-1BB (CD137) and cytokines secreted from Dendritic cells (DCs) such as IL-12, INF-I and IL-15, naïve CD8+ T cells differentiate into cytotoxic T cells (CTLs) [8]. Subsequently, effector CD8+ T cells, which express CXC-chemokine receptor 3 (CXCR3), migrate to tumors from blood and lymphatic systems in response to TH1-type chemokines CX-chemokine ligand 9 (CXCL9), CXCL10 and CXCL11 [9, 10]. Activated CD8+ T cells directly eliminate tumor cells by releasing perforin and granzymes. They induce tumor cell apoptosis by expressing death ligands, mainly Fas ligand (FasL) and TNF-related apoptosis-inducing ligand (TRAIL) [5, 11]. Besides, activated CD8+ T cells secrete multiple cytokines such as INF-γ, TNF-α and TNF-β to regulate and improve overall anti-tumor response, including innate and specific immune systems [12–14].

Mitochondria play crucial and diverse roles during different stages of T cell adaptive immune response [15]. Firstly, as the main organelle for energy supply, mitochondrial energy metabolism provides adenosine triphosphate (ATP) to support all kinds of physiological activities of T cells. Secondly, the subcellular location of mitochondria regulates the directionality of T cell migration. Mitochondria gather near the cell edge where they approach the extracellular chemokines to offer sufficient ATP so that T cells can move towards higher concentrations of chemokines and recruit into tumor sites [15]. As an important signal molecule, the Ca2+ wave, which is prolonged by mitochondria gathering at the immune synapse (IS), is essential for T cell activation [15, 16]. In addition, the low mitochondrial reactive oxygen species (mROS) concentration favors T cell survival and function, and enhances TCR signaling transduction after tumor antigen stimulation in particular [17]. Therefore, the quantity and quality of mitochondria have a significant impact on T cell activity, distribution and function. So, mitochondrial dysfunction would directly affect the anti-tumor effect of CD8+ T cells and promote tumor occurrence and progression. Actually, in chronic infections and cancer, T cells are exposed to continuous antigen stimulation and/or inflammatory signals, and therefore mitochondrial dysfunction is considered a hallmark of resultant deterioration of T cell function, a situation called ‘exhaustion’ [15, 18]. Targeting mitochondria in CD8+ T cells and restoring their original condition to increase CD8+ T cell vitality, or even reverse the dysfunction, may become a feasible treatment strategy for cancer.

In this review, we will introduce the influencing mechanisms of mitochondrial abnormalities on the anti-tumor effect of CD8+ T cells, which may involve mitochondrial energy metabolism, mROS production, mitochondrial dynamic process, CD8+ T cell apoptosis mediated by mitochondria, mitochondrial membrane potential (MMP), and mitochondrial Ca2+ signaling regulation, as well as the possible efficient ways to treat cancer by targeting mitochondria to improve the anti-tumor function of CD8+ T cells.

## Mitochondrial dysfunction affects the anti-tumor effect of CD8+ T cells

**Fig. 1:**
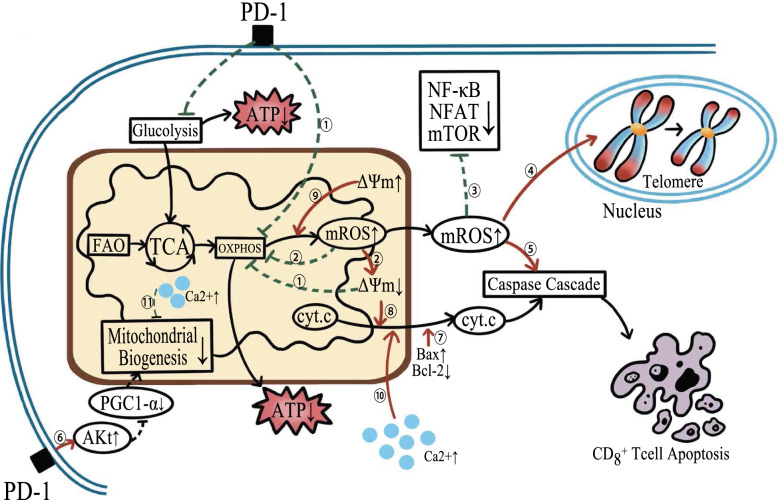
Mechanism of mitochondrial dysfunction in the disturbing anti-tumor activity of CD8+ T cells. (The red arrows indicate promotion, and the green arrows indicate inhibition) ①The decrease of mitochondrial membrane potential and the high expression of PD-1 restrict OXPHOS, reducing ATP synthesis. ②mROS accumulation in turn inhibits OXPHOS or limits respiratory metabolism by decreasing the MMP of CD8+ T cells. ③High level of mROS suppresses CD8+ T cell activation and proliferation by inhibiting NF-κB, mTOR, and NFAT5 signaling pathways. ④mROS accumulation plays a role in attritting telomeres and promoting senescence of CD8+ T cells. ⑤An excessively high mROS concentration activates the caspase signaling cascade and induces CD8+ T cell apoptosis. ⑥PD-1 hinders mitochondrial biogenesis through increasing repression of PGC1-α mediated by Akt. ⑦Imbalance in Bax and Bal-2 accelerates the release of cytochrome c from mitochondria and initiates CD8+ T cell apoptosis. ⑧Low MMP causes a high mitochondrial membrane permeability, triggering the subsequent release of cytochrome c and CD8+ T cell apoptosis. ⑨High MMP leads to a high level of mROS production. ⑩Increased cytoplasmic Ca2+ caused by failed mitochondrial Ca2+ buffering initiates the intrinsic apoptosis process. ⑪Accumulated Ca2+ in matrix inhibits mitochondrial biogenesis

**Fig. 2:**
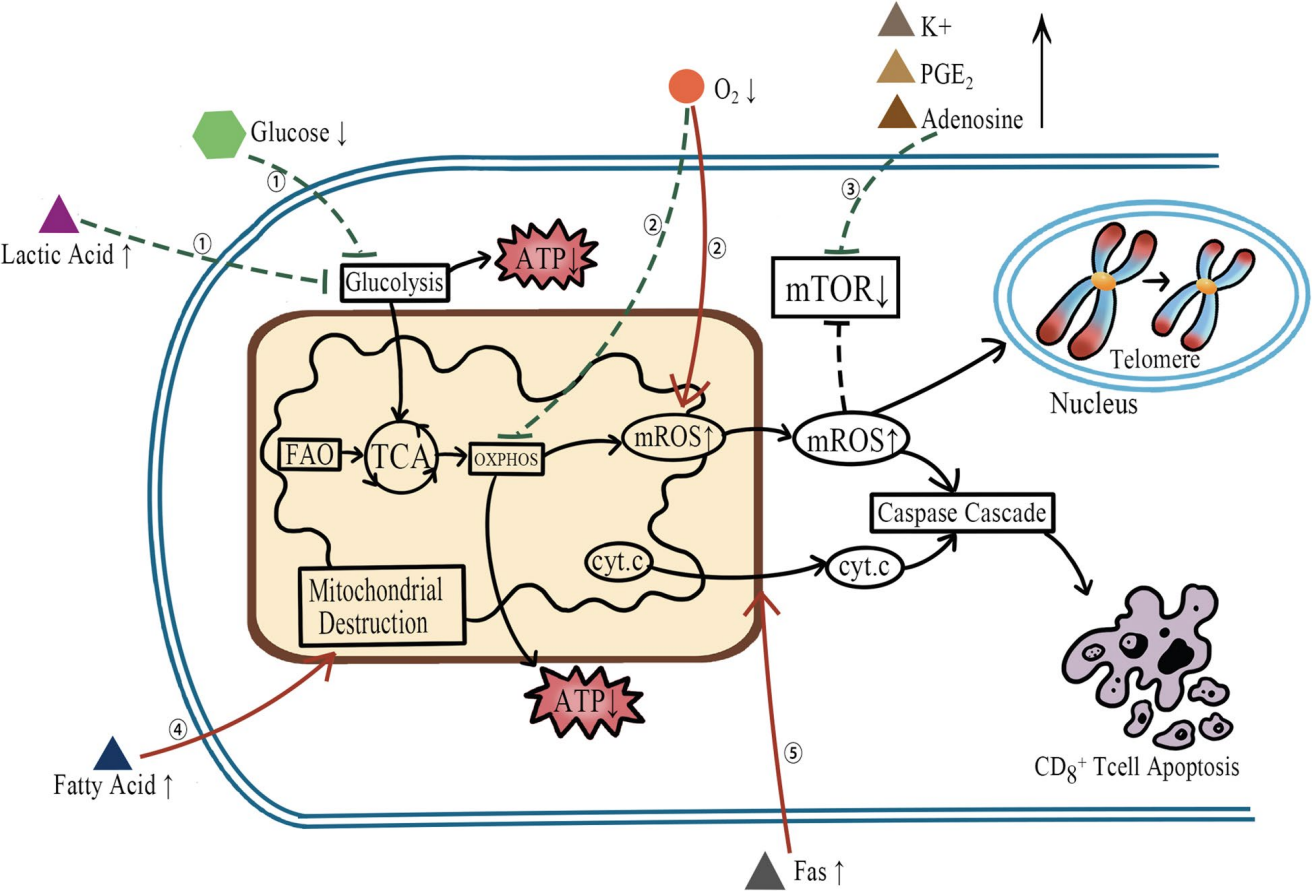
Mechanism of TME affecting the mitochondrial function of CD8+ T cells. (The red arrows indicate promotion, and the green arrows indicate inhibition) ①Glucose competition and lactic acid accumulation increase the glycolysis stress of CD8+ T cells. ②Hypoxia inhibits OXPHOS and increases oxidative stress. ③Released K+ and metabolic waste PEG2, and adenosine interfere with the activity of the mTOR pathway. ④Certain fatty acids can directly damage mitochondrial structures. ⑤Soluble Fas released from tumor cells activates mitochondria-mediated intrinsic apoptotic processes

Mitochondrial physiological activities are critical for the various anti-tumor process of CD8+ T cells, and can even directly impact the number of CD8+ T cells. One strategy for cancer cells to burr the anti-tumor effect of CD8+ T cells is to inhibit their mitochondrial function (Fig. 1 and Fig. 2).

### Mitochondrial energy metabolism maladjustment

CD8+ T cells require a massive amount of energy for normal functioning. Once activated, CD8+ T cells proliferate exponentially, migrate from the original site to tumor locations and synthesize inflammatory cytokines. All these activities require plenty of energy, especially mitochondrial ATP [19, 20] However, it was found in a systemic immunosuppressive property (SIP) tumor model [21] that atypical and non-proteinaceous molecules less than 3 kDa released from SIP-positive tumor cells could weaken mitochondrial energy metabolism and proliferation of CD8+ T cells, thus favoring SIP-positive tumor cells to escape anti-tumor immune response CD8+ T cells.

Naïve T cells depend on oxidative phosphorylation (OXPHOS) and fatty acid oxidation (FAO) for energy, while activated CD8+ T cells more rely on glycolysis even in the presence of sufficient oxygen [19]. This metabolic reprogramming phenomenon is similar to the Warburg effect observed in tumor cells [22]. Therefore, abundant glucose, the substrate for OXPHOS and glycolysis, is the key to CD8+ T cells, whether naïve or active. However, potential competition for glucose and other nutrient substances with tumor cells may inhibit CD8+ T cell functioning, and this metabolic stress can greatly affect signal transduction and gene expression of CD8+ T cells [18, 23–25]. Some in vitro studies [26–28] demonstrated that co-inhibitory molecules programmed cell death protein 1 (PD-1) and cytotoxic T lymphocyte-associated protein 4 (CTLA-4) could attenuate glycolysis in CD8+ T cells and suppress their anti-tumor function. For example, PD-1 can prevent glycolysis by maintaining FAO, and this effect could be achieved by activating the signal transducer and activator of transcription 3 (STAT3) of CD8+ T cells in obese patients [29, 30]. On the other hand, overexpressed PD-1 and CTLA-4 were proved on exhausted CD8+ T cells. So, the deficiency of glucose and energy may exacerbate CD8+ T cell exhaustion.

It cannot be neglectable that OXPHOS is not repressed entirely but continues to increase and plays a role in activated effector CD8+ T cells. Pyruvate, the final product of glycolysis, participates in the tricarboxylic acid cycle (TAC) as a substrate. mROS produced during OXPHOS participates in CD8+ T cell activation by working as an important signal molecule. For instance, it can regulate the nuclear factor of activated T cells (NFAT) to encourage antigen-induced cellular signaling [19, 31]. But studies have found that the level of OXPHOS is very low in tumor-infiltrated CD8+ T cells due to the low mitochondrial mass or MMP [32, 33]. Other studies have also reported that PD-1 engagement could inhibit OXPHOS in human CD8+ T cells through initiating a negative expression of metabolic genes progressively due to seriously impaired mitochondrial cristae [29].

In summary, mitochondrial energy metabolism dysfunction will not only further expand the energy gap of CD8+ T cells but affect the internal signal transduction, eventually resulting in tumor occurrence due to their weakened function in the anti-tumor effect.

### Abnormally high level of mROS

Mitochondria are the major site for intracellular ROS formation [34]. ROS, mainly including superoxide anion (O2-), hydrogen peroxide (H2O2), and hydroxyl radical (OH•), are produced from the partially reduced O2, which renders a higher chemical reaction activity. The conventional view is that ROS are destructive agents, in fact, a suitable threshold level of ROS is necessary for intracellular signal transduction, kinase activation, and biologic responses associated with receptor signaling [35]. A low to moderate level of ROS is crucial for CD8+ T cell activation, proliferation, and effective substance secretion [34]. After the TCR complex binds to the tumor antigen, calcium will influx rapidly, resulting in the increased release of mROS [36]. This released mROS can regulate IL-2 and IL-4 secretion, which are essential for maintaining CD8+ T cell activation [34]. mROS also can increase the phosphorylation of c-Jun N-terminal kinase (JNK) and nuclear factor kappa-B (NF-κB), the levels of transcription factor (TF) STAT-1 and T-bet, as well as the secretion of IL-2, IL-4, TNF-α and granulocyte-macrophage colony stimulating factor (GM-CSF), all of which promote CD8+ T cells to synthesize and secret more INF-γ against tumor cells [34].

When mitochondria dysfunction occurs due to their higher activity, the accumulation of mROS will hurt CD8+ T cells in many ways. Dysfunctional mitochondria produce a large amount of ROS, which will directly cause damage to biological macromolecule DNA, protein and lipid [37]. Overproduction of ROS will inhibit NF-κB phosphorylation and activation, reduce CD8+ T cell sensitivity and response, and decrease the mammalian target activity of rapamycin complex 1 (mTORC1), which is vital in CD8+ T cell activation and metabolism [34, 38]. A high concentration of mROS will accelerate CD8+ T cell senescence. It is proved that mROS is a motivator for telomere length shortening in CD8+ T cells [39]. With reduced calcium influx into mitochondria and increased mROS production, respiration in CD8+ T cells will be depressed. All these alterations can reduce the MMP and slow down ATP synthesis [40]. Long-term exposure to ROS can activate the caspase cascade signal and elevate CD8+ T cell apoptosis in the peripheral tissue, and inhibit NFAT5 by combining with IL-6 promoter and preventing CD8+ T cell expansion [34, 41]. As a result, the number of CD8+ T cells is decreased and the anti-tumor function is aberrated.

A study about clear cell renal cell carcinoma (ccRCC) [42] reported that mitochondrial morphology and energy metabolism were abnormal in ccRCC-infiltrated CD8+ T cells. These condensed and fragmented mitochondria showed a hyperpolarized state and generated a large quantity of ROS. Similarly, abnormal and hyperpolarized mitochondria could also be observed in the peripheral blood of ccRCC patients. These experiments further demonstrated that excessive mROS was attributed to reduced mitochondrial superoxide dismutase 2 (SOD2), which could countervail mROS to relieve oxidative stress, but this protein was measured to be expressed less in ccRCC-infiltrated CD8+ T cells.

In conclusion, elevated mROS derived from stressed mitochondria will expedite functional loss of CD8+ T cells and weaken their anti-tumor effect.

### Disordered mitochondrial dynamic processes

Mitochondrial dynamics mainly refers specifically to mitochondrial fusion and fission, but other dynamic changing processes, including mitochondrial mobility, biogenesis and autophagy, also have an influence on CD8+ T cells [40]. Mitochondrial fusion can promote OXPHOS and FAO in CD8+ T cells by facilitating cristae and electron transport chain (ETC) complex formation as well as increasing substrate intake. Mitochondrial fission can facilitate aerobic glycolysis and promote mitochondrial autophagy to delete dysfunctional mitochondria in CD8+ T cells [40, 43]. More importantly, mitochondrial fragmentation and accumulation underneath the TCR clusters are required for immune synapse formation. At the established IS, mitochondrial fragmentation continues to be needed for ATP supplement, local calcium buffering, TFs activation and cytokine secretion [16, 44]. Mitochondrial mobility determines mitochondrial subcellular localization and local concentrations in CD8+ T cells. Mitochondria gather at the uropod of CD8+ T cells when more energy is required for cell migration [20, 45]. At parallel, mitochondrial fission promotes their own transportation as the smaller mitochondria are more easily to move. In a solid cancer model, CD8+ T cells with defective mitochondrial fission would cause a reduced infiltration [46, 47]. The balance between mitochondrial biogenesis and autophagy sustains a stable scale of CD8+ T cells. Damaged mitochondria demand to be degraded by mitophagy, otherwise, these damaged mitochondria with lower MMP will promote cell senescence [48]. Autophagy related gene 7 (Atg7)-deficient CD8+ T cells are unable to constrain the mitochondrial content, resulting in ROS overproduction and Bcl-2-associated X protein (Bax) elevation, and finally increasing their apoptosis [49].

Previous studies on non-small cell lung cancer (NSCLC) and chronic viral infections reported that high expression of PD-1 on the surface of activated CD8+ T cells restrained transcriptional coactivator peroxisome proliferator-activated receptor-γ coactivator-1α (PGC1-α), a critical gene transcriptional regulator of mitochondrial metabolism and biogenesis, leading to mitochondrial depolarization accompanied with the reduced number and length of cristae, and a trend towards ROS accumulation. CD8+ T cells with these severe functional and structural changes in mitochondria present deficiency in energy metabolism and immune function [26, 29, 50]. Another study [32] demonstrated that PGC1-α-mediated defects in the mitochondrial structure and dynamics were related to chronic protein kinase B (PKB/Akt) activation. PGC1-α is regulated by several signal pathways, among which Akt mediates an obviously suppressive pathway. Compared with resting ones in lymph nodes, CD8+ T cells infiltrating in B16 tumor show a higher level of Akt activation. Consistent with this alteration, Akt-mediated inhibitory forkhead box O (Foxo) phosphorylation is increased as well.

Therefore, abnormal mitochondrial dynamics will cause abnormal structural and functional changes in mitochondria, which further blunts the anti-tumor response of CD8+ T cells, thus promoting tumor progression.

### Increased mitochondria-mediated intrinsic apoptosis

Mitochondrial-mediated intrinsic apoptosis is an important pathway for programmed death in eukaryotic cells. According to the classical cell theory, mitochondrial membrane permeability will increase when cell apoptosis launches, resulting in the release of cytochrome c, apoptosis-inducing factor (AIF), endonuclease G and other nucleases and proteases from mitochondria into the cytoplasm [51]. This process can be regulated by B-cell lymphoma-2 (Bcl-2) family proteins [52]. Released cytochrome c combines with apoptotic protease-activating factor 1 (Apaf-1), caspase-9 and ATP, forming apoptosomes. Apoptosomes can activate caspase-9 and trigger subsequent caspase cascade, inducing the degradation phase of apoptosis [53]. In the meantime, endonuclease G and AIF lead to nuclear DNA degradation in a caspase-independent manner [54]. The increased apoptosis of CD8+ T cells mediated by mitochondria and the decreased amount of CD8+ T cells will inevitably reduce the immune surveillance function, which provides a chance for tumor development.

In patients with squamous cell carcinoma of the head and neck (SCCHN), the inhibited function of tumor-infiltrated CD8+ T cells was possibly caused by an increased proportion of apoptosis, which is visible both at the tumor sites and in the peripheral blood [55–57]. The same phenomenon was also observed in patients with other cancer types [58, 59]. Studies have demonstrated that there is an imbalance between pro-apoptosis and anti-apoptosis proteins in the Bcl-2 family in SCCHN CD8+ T cells. Compared with the control group, the expression level of Bax and Bcl-XL in the SCCHN group was upregulated markedly while the expression of Bcl-2 remained unchanged [55], suggesting that the mitochondria-mediated intrinsic apoptosis pathway may participate in SCCHN CD8+ T cell death in the peripheral blood. When mitochondrial Bcl-2 was downregulated, activated CD8+ T cells cultured in vitro quickly transformed into an apoptotic state, probably due to lack of IL-2, a crucial factor for maintaining an appropriate level of intracellular Bcl-2 and CD8+ T cell survival [60].

Therefore, an increased signal for inducing apoptosis in the extracellular environment and consequent enhanced intrinsic apoptosis mediated by mitochondria would decrease the number of CD8+ T cells involved in the anti-tumor response, thus promoting tumor development.

### Mitochondrial membrane potential disruption

Stable MMP is essential for mitochondria to maintain their normal structure and physiological function. MMP is generated by proton pumps (ETC complex I, III and IV), by which, protons can be actively transported from inside to outside the inner membrane of mitochondria. The accumulation of a large number of protons in the intermembrane space eventually creates an electrochemical gradient with negative charges inside the mitochondrial matrix [61].

Data from different human CD8+ T cells suggest that tumor-infiltrated CD8+ T cells display an improvement in metabolism as represented by the increase in the MMP and mitochondrial number [62]. MMP is not only involved in ATP production, Ca2+ uptake and storage, ROS generation and decomposition, but is also closely related to cytochrome c release and cell apoptosis [63]. It was found that co-culture of CD8+ T cells with tumor cells decreased or even abolished the MMP of CD8+ T cells, further leading to an accumulation of dysfunctional mitochondria with disrupted membrane structures, cristae structure and declined cristae number and length of crista [64]. One of the reasons may be the exposure to gangliosides GD3 released from the tumor cells [65–67]. GD3 has been proven to be a selective inducer of the mitochondrial permeability transition pore (mPTP) in situ [68]. When mPTP opens, protons gathered outside the inner mitochondrial membrane start to flow inward driven by an electrochemical gradient, resulting in the MMP loss. Destructed structure and increased permeability of the inner mitochondrial membrane allow H2O2 and some solute molecules to influx freely, which further leads to mitochondrial swelling and outer mitochondrial membrane rupture. Eventually, cytochrome c is released and the caspase cascade is activated. CD8+ T cells subsequently began apoptosis processing [69].

However, some studies argued that CD8+ T cells with lower MMP had a longer survival time in vivo and a better anti-tumor effect [70]. They believed that lower MMP was directly associated with less ROS, which supports DNA protection and reparation. Compare with CD8+ T cells with high MMP, CD8+ T cells with low MMP own an extensive storage capacity of oxidized glutathione (GSSG) as well as an over expression of ROS-detoxifying enzymes mRNAs such as catalase (Cat), glutathione peroxidase 4 (Gpx4), superoxide dismutase 1 (SOD1) and SOD2. These enzymes are conductive to maintain intracellular redox equilibrium in CD8+ T cells [70]. The same conclusion can be proved in chronic lymphocytic leukemia (CLL) patients. CLL-derived CD8+ T cells showed a high MMP when the genes encoding subunits of ETC complexes I, III, IV and V, and mitochondrial-associated protein phosphatase 2 were expressed at a consistently high level, and the fitness of these CD8+ T cells was damaged [71].

In conclusion, impaired MMP will seriously affect the function and survival of CD8+ T cells in favor of tumor growth.

### Fluctuant Ca2+ signaling regulates mitochondria in CD8+ T cells

Ca2+, a second messenger in CD8+ T cells, has been proved to be involved in multiple signaling pathways to regulate a variety of cellular and mitochondrial physiological processes [72]. Upon antigen stimulation, rising cytosolic Ca2+ binds with calmodulin (CaM) to activate protein phosphatase calcineurin, which later phosphorylates NFAT, resulting in IL-2 production and continuous cell proliferation [31, 73]. Ca2+ regulates the energy metabolism of CD8+ T cells in many aspects. At first, Ca2+ can activate three rate-limiting enzymes in TAC [pyruvate dehydrogenase (PDH), ​​​​​NAD+ isocitrate dehydrogenase (NAD-IDH) and α-ketoglutarate dehydrogenase (α-KGDH)], leading to more abundant ATP supply [74]. Secondly, Ca2+ regulates glycolysis in activated T cells by controlling the expression of glucose transporters (GLUT-1 and GLUT-3) and some crucial TFs [c-Myc, interferon regulatory factor 4 (IFR-4) and hypoxia inducible factor 1 (HIF-1)] [75]. Ca2+ also can enhance FAO via activating adenosine 5′-monophosphate (AMP)-activated protein kinase (AMPK) [72]. Extracellular Ca2+ influx after CD8+ T cell activation induces the rearrangement of the cytoskeleton, favoring the interactions between the TCR complex and APCs [76]. And the cytotoxicity of TILs is reported to rely on Ca2+ concentration [77, 78]. In addition, Ca2+ is a key factor in the activation of phosphoinositide 3-kinase (PI3K)-AKT-mTORC1 signaling pathway, further regulating mRNA transcription, protein synthesis and cell differentiation [72, 75].

Upon CD8+ T cells activation, phosphatidylinositol biphosphate (PIP2) is hydrolyzed in inositol triphosphate (IP3) and diacyl glycerol (DAG) by phospholipase C (PLC). Then, IP3 binds to the IP3 receptor (IP3R) on the endoplasmic reticulum (ER) to release stored Ca2+ into the cytoplasm [79]. Free Ca2+ decreased in ER will trigger the opening of Ca2+ release-activated Ca2+ channel (CRAC) on cytomembrane, leading to a further increased level of cytosolic Ca2+ by store-operated Ca2+ entry (SOCE) [80]. However, Ca2+ accumulation is not always beneficial to cells, so mitochondria, also as known as the main intracellular Ca2+ storage organelle, play an essential role in the Ca + buffering process. Mitochondria redistribute to the vicinity of the IS in a microtubule-actin-dependent manner [81], where they uptake Ca2+ through the voltage dependent anion channel (VDAC) in the outer mitochondrial membrane and the mitochondrial Ca2+ uniporter (MCU) in the inner mitochondrial membrane [82]. This reaction effectively prevents CRAC inhibition by Ca2 + −dependent negative feedback [72]. On the other hand, Ca2+ in mitochondria can be slowly extruded into the cytoplasm through mitochondrial Na+/Ca2+ exchanger (NCLX) and Na + independent exchanger mPTP, helping to prolong the Ca2+ signaling as well [76]. Mitochondrial Ca2+ buffering also can avoid the modulation of plasma membrane Ca2+ ATPase (PMCA), which pumps Ca2+ out of T cells, and maintain an appropriate intensity of SOCE [83].

In view of the complexity and importance of Ca2+ signaling, the fluctuant Ca2+ signaling related to mitochondria will inevitably disturb the anti-tumor function of CD8+ T cells. Continual Ca2+ extrusion due to the increased mPTP permeability will cause mitochondrial matrix swelling and outer membrane rupture, resulting in the release of cytochrome c and apoptosis proteins [84]. Because of the failed Ca2+ buffering of depolarized mitochondria, excessive Ca2+ in cytoplasm will inhibit CRAC activation, and even trigger the intrinsic apoptosis pathway. In this situation, Mfn2 has been reported to restrain partial Ca2+ influx to protect cells [85]. Similarly, MCU deletion also will damage mitochondrial Ca2+ uptake, as well as mitochondrial function, such as OXPHOS and mROS production [72]. In addition, mitochondrial Ca2+ overload due to the extra influx or hindered extrusion (e.g. deficiency of Na + or NCLX) can not only influence mROS production, but also mitochondrial biogenesis, which might through altering PGC1-α expression or Drp-1 phosphorylation [72, 83, 86].

Existing evidence has confirmed that a microenvironment with low Ca2+ concentration is more favorable for TILs. A clinical study on NSCLC treated with nivolumab has found that low serum Ca2+ level was parallel to significantly prolonged overall survival (OS) and progression free survival (PFS) [87]. Indeed, the optimum intracellular and extracellular Ca2+ for TILs were respectively defined as 122-334 nmol/L and 23-625 μmol/L, the latter being much lower than the normal physiological concentration [78]. These results suggest properly restraining the Ca2+ signaling by decreasing the extracellular Ca2+ or selectively blocking CRAC to prevent CD8+ T cell exhaustion and enhance the efficacy of TILs [78, 88]. However, regrettably, none of the above studies addressed the potential role of mitochondria.

Although we can safely draw a conclusion that mitochondrial Ca2+ regulation is important to the anti-tumor function of CD8+ T cells, there are not many specific types of research that have focused on this. So, further investigation is required to address this issue.

### Mitochondria of CD8+ T cells in the tumor microenvironment

The tumor microenvironment (TME), composed of cellular components (tumor cells, immune cells and other stromal cells) and non-cellular components (extracellular matrix, chemokines, cytokines and growth factors), has a complex effect on anti-tumor immunity and cancer fate [89]. In severe hypoxic TME, CD8+ T cells are under high oxidative stress conditions. Dysfunctional mitochondria generate unbearable mROS, maintaining continuous activation of NFAT, which upregulates transcriptional factors TOX and NR4A to induce the expression of exhaustion-related genes [90, 91]. Hypoxic interferes with mitochondrial fusion through the miR24-Myc-Mfn1 axis. Decreased OXPHOS in fragmented mitochondria cannot provide sufficient ATP for CD8+ T cells [92]. Hypoxia inducible factor-1 (HIF-1) also disturbs OXPHOS by activating pyruvate dehydrogenase kinase 1 (PDK1). In the meantime, HIF-1 activates mTORC1 and lactate dehydrogenase A (LDHA) to enhance glycolysis, but this exacerbates the glucose deficiency in TME [93, 94]. On the one hand, the intense glucose competition in TME limits CD8+ T cells’ energy support [95], and on the other hand, the accumulation of metabolism wastes and negative factors from tumor cells can damage the mitochondria of CD8+ T cells. The vigorous aerobic glycolysis in tumor cells produces massive lactic acid, leading to adverse TME with a low pH, in which glycolysis of CD8+ T cells is declined for inhibited polyphosphate kinase (PPK) [96]. Accumulated adenosine and prostaglandin E2 (PGE2) in TME respectively bind to A2A and EP4 receptors on CD8+ T cells, causing increased activity of protein kinase A (PKA), which negatively regulates mTORC1, the important pathway for mitochondrial function [95, 97, 98]. Dead tumor cells will release abundant K+ into TME. High K+ mediates Akt-mTORC1 inactivation via protein phosphatase 2A (PP2A), with possible involvement of zinc finger proteins 91 (ZFP91) [99, 100]. Excessive lipids in TME, particularly oxidized lipids and some certain long-chain fatty acids, can directly destroy mitochondria structure behind transferred into CD8+ T cells [101, 102]. Another immunosuppressive metabolite is D-2-hydroxyglutarate (D2HG), and it can inhibit ATP synthase located on the inner mitochondrial membrane [103]. Additionally, soluble Fas secreted from tumor cells also can activate mitochondria-mediated intrinsic apoptosis, depressing anti-tumor immunity of CD8+ T cells [104]. At last, sustained antigenic stimulation, severe hypoxia, nutritional deficiencies, acidic environment, cholesterol accumulation, high concentration of K+ and soluble factors [such as vascular endothelial growth factor A (VEGF-A), indoleamine2,3-dioxygenase1 (IDO), INF-γ and TGF-β] secreted by other cells in TME all upregulate expression of co-inhibition receptors (mainly PD-1 and CTLA-4) on CD8+ T cells. They can affect mitochondrial function through multiple pathways [91, 105].

Recent single-cell transcriptome results have revealed some differentially expressed genes that may be associated with dysfunctional mitochondria of exhausted CD8+ T cells in TME. SARDH, NDUFB3 and HSPA1A are involved in the ETC, involving in ATP metabolism and mROS production [106, 107]. FABP5 and TPI1 are implicated in multiple energy metabolic processes, including glucose metabolism (both OXPHOS and glycolysis) and fatty acid metabolism [106, 107]. FABP5 may also directly shape mitochondrial cristae [108]. CD38 not only negatively affects metabolism, but also activates the mitochondria-mediated intrinsic apoptosis [107]. Another two genes PHLDA1 and EPSTI1 facilitate intrinsic apoptosis as well [107, 109]. PRDX3 and HSBP1 can reduce oxidative stress levels and maintain a stable MMP [107]. In addition to cell apoptosis and electron transport, CDK1 alters mitochondrial dynamics by regulating Drp1 [110, 111].

In the meantime, several key TFs were discovered. STAT2 regulates mitochondrial fission, while STAT3 regulates mitochondrial Ca2+ homeostasis through the ETC [112, 113]. FOXP1, FOXP3, FOXO1 and GATA3 all control mitochondrial respiration and oxidative stress as well as mitochondrial dynamics, autophagy and biogenesis [112, 114–116]. NF-Κb and JUN are mainly associated with mitochondrial fragmentation and increased cellular apoptosis [112]. Besides, PRDM1, which encodes protein blimp1, is proved as an important TF connected with PGC1-α-mediated mitochondrial biogenesis [117]. Other TFs, like BATF, VHL and ETV1, respectively regulate mitochondrial energy metabolism and intrinsic apoptosis process [115, 118, 119].

Thus, it can be seen that TME mainly affects mitochondrial metabolism, oxidative stress, intrinsic apoptosis and dynamic processes in CD8+ T cells.

## Treatment of cancer by targeting mitochondria in CD8+ T cells

**Fig. 3:**
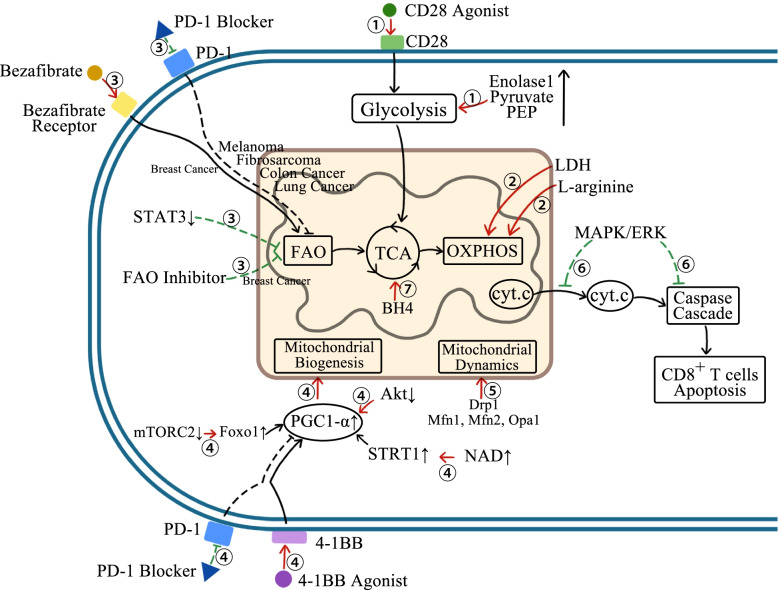
Targeting multiple metabolic pathways of mitochondria can enhance the anti-tumor effect of CD8+ T cells. (The red arrows indicate promotion, and the green arrows indicate inhibition) ①Activating CD28, increasing enolase 1 activity, or adding exogenous pyruvate and PEP can promote the glycolysis of CD8+ T cells. ②Inhibiting LDH activity and supplementing exogenous L-arginine can promote OXPHOS of CD8+ T cells. ③For melanoma, fibrosarcoma, colon cancer and lung cancer, a combination of bezafibrate with PD-1 blockade can promote FAO and function of CD8+ T cells, while inhibiting FAO can improve the anti-tumor effect of CD8+ T cells in breast cancer. ④Up-regulating the expression level of transcriptional activity of PGC1-α in CD8+ T cells can facilitate mitochondrial biogenesis and functional recovery. It can be achieved by stimulating 4-1BB combined with PD-1 blockade, inhibiting the activity of Akt, inhibiting the mTORC2 pathway to activate Foxo1, and using nicotinamide adenine dinucleotide to activate SIRT1. ⑤Regulating mitochondrial fusion and fission by essential GTPase (Drp1, Mfn1, Mfn2 and Opa1) can control CD8+ T cell function. ⑥MAPK/ERK can prevent the release of cytochrome C from mitochondria to cytoplasm and the activation of caspase cascade, decreasing CD8+ T cell apoptosis. ⑦Addition of BH4 can regulate mitochondrial iron transport and respiration in CD8+ T cells, increasing the activation of CD8+ T cells

Although mitochondrial abnormality and dysfunction can accelerate the loss of the anti-tumor effect of CD8+ T cells, this consequence is not irreversible. We can aim at mitochondria in CD8+ T cells as a target, and recover the anti-tumor activity of CD8+ T cells by regulating the mitochondrial metabolism, dynamics or other physiological processes, and increasing mitochondrial mass and quality (Fig. 3). It may prove to be a new strategy for cancer treatment in the future.

### Targeting mitochondrial energy metabolism of CD8+ T cells

Glycolysis provides the most fuel for activated CD8+ T cells. It was reported [120] that CD28 co-stimulation could enhance glycolysis of ccRCC-infiltrated CD8+ T cells and recover their metabolism mainly by increasing glucose transporter 3 (GLUT3), and under CD28 co-stimulation mitochondria of CD8+ T cell showed more fusion and quantity, boosting mitochondrial activity restoration. Another study [33] reported that enolase 1 activity was downregulated in metabolism-impaired tumor-infiltrated CD8+ T cells. It restricted the production of phosphoenolpyruvate (PEP), which is known to play an important role in glycolysis as an intermediate, as well as production of pyruvate, which is a downstream product of enolase 1. So, increasing enolase 1 activity or adding exogenous PEP and pyruvate is effective for repairing partial glucose metabolism and anti-tumor activity of CD8+ T cells.

Regulating mitochondrial OXPHOS is another means to regulate CD8+ T cell activity. Inhibition of lactate dehydrogenase (LDH) improves pyruvate oxidation, promotes pyruvate into TAC and finally enhances OXPHOS, and the effect is much more efficient with IL-2 synergy [121]. Lots of L-arginine is consumed by activated CD8+ T cells for downstream metabolism, so the intracellular level of L-arginine is significantly reduced inside activated CD8+ T cells. Some groups [122] demonstrated that adding exogenous L-arginine could upregulate the serine biosynthesis pathway, and further strengthen TAC, leading to the transformation of CD8+ T cell metabolism from glycolysis to OXPHOS, which reduces the Warburg effect, and improves CD8+ T cell survival and anti-tumor function as well.

Tumor-infiltrated CD8+ T cells have to experience nutrient limitations due to the intense competition with cancer cells for oxygen, glucose and other key nutrients. This kind of nutrient restriction can cause hyporesponsiveness of CD8+ T cells even under stimulation by highly antigenic tumor cells [123]. In melanoma immunotherapy, enhancing FAO could compensate for the energy deficiency of CD8+ T cells and elevate the anti-tumor effect [124]. More studies in fibrosarcoma, colon cancer and lung cancer mouse models demonstrated that combined treatment by benzabate and PD-1 blockage could activate mitochondria of CD8+ T cells and promote FAO to provide extra energy. Besides, the same treatment also worked through other pathways by ①improving CD8+ T cell glycolysis and OXPHOS; ②upregulating Bcl-2 and carnitine palmitoyl transferase 1 (Cpt1), which can bind together to decrease CD8+ T cell apoptosis, and the latter was also found to participate in FAO; ③promoting CD8+ T cell infiltration by increasing the expression of CXCL9 and CXCL10 from tumor tissues and CXCR3 in CD8+ T cells [125, 126].

So far, it is still difficult to draw a conclusion on whether enhancing FAO is always positive for CD8+ T cells in different types of cancers at present. A study [30] about obesity-associated breast cancer reported that suppressed FAO could recover glycolysis and the anti-tumor capacity of CD8+ T cells. Given the role of obesity is complex in cancer progression and immunity regulation, we won’t discuss it further in this article. In all, the above facts indicate that targeting mitochondrial energy metabolism can regulate the anti-tumor activity of CD8+ T cells.

### Targeting mitochondrial dynamic of CD8+ T cells

PGC1-α is an essential transcriptional co-activator that regulates mitochondrial biogenesis by binding to the nuclear receptors and specific sequences in the promoter region of the target genes [127]. The level of PGC1-α depends on energy demand [128]. With CD8+ T cell activation, the PGC1-α expression is upregulated, while post-translational modification can modulate the transcriptional activity of PGC1-α [127]. Phosphorylation of PGC1-αby Akt will largely inhibit its transcriptional activity [128, 129]. As we mentioned previously, abnormal mitochondrial dynamics in tumor-infiltrated CD8+ T cells are related to the downregulation or inhibition of PGC1-α. Thus, increasing PGC1-α expression and activity may be able to rescue mitochondria and the anti-tumor function of CD8+ T cells.

CD8+ T cells with PGC1-α overexpression showed a higher OXPHOS level and a greater spare respiratory capacity (SRC) in vitro, suggesting that their mitochondrial biogenesis is restored. In line with this, these CD8+ T cells also exhibited a longer survival time and an increased anti-tumor effect [32]. The PGC1-α level was low in CD8+ T cells with overexpressed PD-1. Although mitochondrial activity was improved in tumor-bearing mice after the PD-1 block, simply blocking PD-1 could not completely reverse mitochondria dysfunction [21, 32, 130]. Another costimulatory molecule 4-1BB, highly expressed on exhausted T cells, was also found to elevate PGC1-α-mediated mitochondrial biogenesis and fusion by activating the p38- mitogen-activated protein kinase (MAKP) signal pathway. A combination of 4-1BB agonism might help overcome the metabolic dilemma of CD8+ T cells. In addition, 4-1BB co-stimulation combined with PD-1 block could exert a potent anti-tumor effect [40, 130]. Akt-mediated signaling activation repressed the PGC1-α in CD8+ T cells [32]. Akt inhibition helped improve FAO, SRC, as well as survival and expansion of transferred anti-tumor CD8+ T cells in a study about adoptive cell therapy [131]. Although they did not further explore whether the effect had a direct link to PGC1-α, their finding could not be excluded as a possible mechanism. In addition, PGC1-α is also regulated by Foxo1. Inhibition of the mTORC2 pathway to prevent Fxoxo1 phosphorylation and activation could promote PGC1-α and subsequent generation of memory CD8+ T cells [49, 132, 133]. Mouse embryonic fibroblasts treated with nicotinamide adenine dinucleotide (NAD) could deacetylate PGC1-α by activating silent information regulator type 1 (SIRT1), consequently increasing the PGC1-α transcriptional activity and restoring the mitochondrial original function [134–136]. In mice fed with nicotinamide riboside (NR), the precursor of NAD generation, melanoma and colon tumors growth was significantly restricted by CD8+ T cell-dependent immune. In vitro, CD8+ T cells treated with NR showed a strong anti-tumor response for reduced mROS generation and more secretion of effector cytokines. But these results were more likely to be related to enhanced autophagy of dysfunctional mitochondria than mitochondrial biogenesis [64]. As the complex regulation of the mitochondrial dynamic process, more studies are required for the specific mechanism.

Besides mitochondrial biogenesis, the anti-tumor ability of CD8+ T cells can be changed through regulating mitochondrial fusion and fission. Several GTPases belonging to the Dynamin family are the core components in mitochondrial dynamics. GTPase Dynamin-related protein 1 (Drp1) oligomerization drives mitochondrial fission. Mitofusins 1 (Mfn1) and Mfn2 actuate outer mitochondrial membrane fusion, followed by inner mitochondrial membrane fusion actuated by optic atrophy 1 (Opa1) [137, 138]. These proteins are essential targets to control mitochondrial dynamics as well.

It is observed that drugs promoting mitochondrial fusion extended the longevity of effector CD8+ T cells and enhanced their secretion of INF-γ and TNF-α in tumor-bearing mice [43]. Mitochondrial fusion recovers cellular function through increasing mitochondrial metabolism and decreasing the sensitivity of cell death signals [139]. Several molecules can directly modulate mitochondrial fusion, such as SAMβA (preventing Mfn 1 phosphorylation) [140]; 15-oxospiramilactone (preventing degradative mitofusins ubiquitination) [141]; Leflunomide (facilitating mitofusins expression) [142]. While other molecules can enhance mitochondrial fusion by inhibiting fission, such as P110 and P259 (blocking Drp1 receptor) [143, 144]; mdivi-1 (inhibiting Drp1 GTPase activity) [145]. But at the same time, we must realize that promoting mitochondrial fusion is not always beneficial to CD8+ T cells. Drp1-mediated mitochondrial fission not only helps chemotaxis of CD8+ T cells and their infiltration into the tumor site but controls mitochondria distribution during cell expansion. It is proved that CD8+ T cell migration and effectors function were hindered when Drp1 expression was inhibited [146]. Therefore, enhancing Drp1-mediated mitochondrial fission may promote CD8+ T cell infiltration towards the tumor, particularly in the absence of effector CD8+ T cells [20, 45, 147]. On the other hand, increased Drp-1-mediated mitochondrial fission has been confirmed to promote aerobic glycolysis, which activated CD8+ T cells relied on [43]. However, there are only a few reports about mitochondria-fission-promoting drugs. This is an area worth exploring in the future.

Since mitochondrial fusion and fission can both help the anti-tumor function of CD8+ T cells, how do we choose or balance between these two seemingly contradictory options in practical application? Based on the current present evidence, mitochondrial fission contributes more to CD8+ T cells activation and infiltration, while mitochondrial fusion is connected to cellular longevity and cytokine secretion after cell activation, so a dynamic therapeutic strategy that promotes mitochondrial fission at the early stage and promotes mitochondrial fusion later and is continuously adjusted is needed.

### Targeting mitochondria-mediated intrinsic apoptosis of CD8+ T cell

Interaction between extracellular death ligands and superficial death receptors results in sequential activation of Fas-associated death domain (FADD) protein and caspase-8 in CD8+ T cells. Activated caspase-8 splits Bid protein to cleaved Bid (tBid), which translocates onto mitochondria and causes mitochondrial depolarization. Then, MMP is changed and the mitochondrial membrane becomes more permeable, allowing cytochrome c release [148–150]. Mitogen-activated protein kinase (MAPK)/extracellular signal-regulated kinase (ERK) can negatively regulate apoptosis by inhibiting caspase-8 and Bit activation, impeding mitochondrial depolarization, and cytochrome c release, protecting CD8+ T cells from death [149, 151, 152].

### Targeting other mitochondrial physiological processes in CD8+ T cells

Tetrahydrobiopterin (BH4) is required for CD8+ T cell expansion both in vitro and in vivo, because it is involved in iron metabolism and mitochondrial respiration regulation. After BH4 inhibition by kynurenine, the anti-tumor function of CD8+ T cells is suppressed, and this phenomenon may be mechanistically associated with disturbed iron redox of cytochrome c in mitochondria and resultant mitochondrial dysfunction. Therefore, increasing the BH4 level can to some extent help CD8+ T cells get out of this dilemma and restrain tumor development [153].

## Conclusion

In this review, we have discussed the adverse effects of mitochondrial dysfunction on CD8+ T cells, and systematically reviewed the recent research progress of targeting mitochondria to restore the anti-tumor function of CD8+ T cells. Beyond the classical function of energy metabolism and ATP production, we also emphasized the important role of mitochondria in intracellular signal transduction, cell activation and migration, cell senescence and apoptosis and other crucial physiological processes in CD8+ T cells. In particular, single-cell sequencing techniques have unveiled some possible genes with altered expression in the TME, providing additional clues to delve into the mechanism of mitochondrial dysfunction of CD8+ T cells. So, it is important to recognize that mitochondrial dysfunction can disturb the original normal immune function of CD8+ T cells by various means, further leading to tumor occurrence and progression. On the one hand, the complex relationship between tumor growth and anti-tumor immunity has been confirmed again, but on the other hand, several possible treatment targets of mitochondria are revealed at the same time, which may reverse the depressed anti-tumor function of CD8+ T cells.

Prospectively, targeting mitochondria to enhance the anti-tumor function of CD8+ T cells presents a better further application perspective in tumor immunotherapy, especially in chimeric antigen receptor T cell (CAR-T) therapy. CAR-T cells (mainly CD8+ T cells) can specifically recognize tumor antigens in vivo and eliminate tumor cells through cellular immunity. But the efficacy of CAR-T therapy in solid tumors is not satisfactory as that in hematological malignancies. CAR-T cell administration is found to show an impaired anti-tumor effect in vivo (the specific manifestations are limited proliferation, hindered migration and infiltration, transient persistence, premature senescence as well as TME immunosuppressive) [154, 155], an important reason for which is cellular mitochondrial dysfunction in the TME [156, 157]. Therefore, restoring mitochondrial function may be able to help break the dilemma and improve the prognosis of cancer patients. Nonetheless, the treatment strategy of targeting mitochondria to regulate the anti-tumor function of the immune system is still in the early stage. More studies are needed to evaluate the efficacy and safety of this approach in clinical use.

## Data Availability

Not applicable.

## Abbreviations

CD: Cluster of differentiation
APCs: Antigen-presenting cells
TCR: T cell antigen receptor
CTLs: Cytotoxic T cells
ATP: Adenosine triphosphate
IS: Immune synapse
mROS: Mitochondrial reactive oxygen species
MMP: Mitochondrial membrane potential
MHC: Major histocompatibility complex
IL: Interleukin
DCs: Dendritic cells
CXCXR: CXC-chemokine receptor
TH: T helper
CXCL: Chemokines CX-chemokine ligand
pMHC: Peptide-loaded major histocompatibility complex
MTOC: Microtubule-organizing center
FasL: Fas ligand
TNF: Tumor necrosis factor
TRAIL: TNF-Related apoptosis-inducing ligand
INF: Interferon
ADCC: Antibody- dependent cellular cytotoxicity
TNFR: TNF receptor
SIP: Systemic immunosuppressive property
OXPHOS: Oxidative phosphorylation
FAO: Fatty acid oxidation
CTLA: Cytotoxic T lymphocyte-associated protein
PD: Programmed cell death protein
STAT: Signal transducer and activator of transcription
TAC: Tricarboxylic acid cycle
NFAT: Nuclear factor of activated T cells
JNK: c-Jun N-terminal kinase
NF-κB: Nuclear factor kappa-B
TF: Transcription factor
Gm-csf: Granulocyte-macrophage colony stimulating factor
mTORC: Mammalian target activity of rapamycin complex
ccRCC: Clear cell renal cell carcinoma
SOD: Mitochondrial superoxide dismutase
ETC: Electron transport chain
Atg: Autophagy related gene
Bcl-2: B-cell lymphoma-2
Bax: Bcl-2-associated X protein
NSCLC: Non-small cell lung cancer
PGC1-α: Peroxisome proliferator-activated receptor-γ coactivator-1α
PKB/Akt: Chronic protein kinase B
Foxo: Forkhead box O
AIF: Apoptosis-inducing factor
Apaf-1: Apoptotic protease-activating factor 1
SCCHN: Squamous cell carcinoma of the head and neck
mPTP: Mitochondrial permeability transition pore
GSSG: Glutathione
Cat: Catalase
Gpx: Glutathione peroxidase
SOD: Superoxide dismutase
CLL: Chronic lymphocytic leukemia
Glut: Glucose transporter
PEP: Phosphoenolpyruvate
LDH: Lactate dehydrogenase
Cpt: Carnitine palmitoyl transferase
SRC: Pare respiratory capacity
MAKP: Mitogen-activated protein kinase
NAD: Nicotinamide adenine dinucleotide
SIRT: Silent information regulator type
NR: Nicotinamide riboside
Drp: Dynamin-related protein
Mfn: Mitofusins
Opa: Optic atrophy
FADD: Fas-associated death domain
MAPK: Mitogen-activated protein kinase
ERK: Extracellular signal-regulated kinase
CAR-T: Chimeric antigen receptor T cell
CaM: Calmodulin
PDH: Pyruvate dehydrogenase
NAD-IDH: NAD+ isocitrate dehydrogenase
α-KGDH: α-ketoglutarate dehydrogenase
GLUT: Glucose transporters
IFR-4: Interferon regulatory factor 4
HIF-1: Hypoxia inducible factor 1
AMP: Adenosine 5’-monophosphate
AMPK: AMP-Activated protein kinase
PI3K: Phosphoinositide 3-kinase
PIP2: Phosphatidylinositol biphosphate
IP3: Inositol triphosphate
DAG: Diacyl glycerol
PLC: Phospholipase C
IP3R: IP3 receptor
ER: Endoplasmic reticulum
CRAC: Ca2+ release-activated Ca2+ channel
SOCE: Store-operated Ca2+ entry
VDAC: Voltage dependent anion channel
MCU: Mitochondrial Ca2+ uniporter
NCLX: Na+/Ca2+ exchanger
PMCA: Plasma membrane Ca2+ ATPase
OS: Overall survival
PFS: Progression free survival
TME: Tumor microenvironment
PDK1: Pyruvate dehydrogenase kinase 1
LDHA: Lactate dehydrogenase A
PPK: Polyphosphate kinase
PGE2: Prostaglandin E2
PKA: Protein kinase A
PP2A: Protein phosphatase 2A
ZFP91: Zinc finger proteins 91
D2HG: D-2-hydroxyglutarate
VEGF-A: Vascular endothelial growth factor A
IDO: Indoleamine2, 3-dioxygenase1
